# The application of artificial intelligence in diabetic retinopathy: progress and prospects

**DOI:** 10.3389/fcell.2024.1473176

**Published:** 2024-10-25

**Authors:** Xinjia Xu, Mingchen Zhang, Sihong Huang, Xiaoying Li, Xiaoyan Kui, Jun Liu

**Affiliations:** ^1^ Department of Radiology, The Second Xiangya Hospital of Central South University, Changsha, China; ^2^ Beijing Tongren Hospital, Capital Medical University, Beijing, China; ^3^ School of Computer Science and Engineering, Central South University, Changsha, Hunan, China; ^4^ Clinical Research Center for Medical Imaging in Hunan Province, Changsha, China; ^5^ Department of Radiology Quality Control Center in Hunan Province, Changsha, China

**Keywords:** artificial intelligence, diabetic retinopathy, diagnosis, prospects, images, molecular marker

## Abstract

In recent years, artificial intelligence (AI), especially deep learning models, has increasingly been integrated into diagnosing and treating diabetic retinopathy (DR). From delving into the singular realm of ocular fundus photography to the gradual development of proteomics and other molecular approaches, from machine learning (ML) to deep learning (DL), the journey has seen a transition from a binary diagnosis of “presence or absence” to the capability of discerning the progression and severity of DR based on images from various stages of the disease course. Since the FDA approval of IDx-DR in 2018, a plethora of AI models has mushroomed, gradually gaining recognition through a myriad of clinical trials and validations. AI has greatly improved early DR detection, and we’re nearing the use of AI in telemedicine to tackle medical resource shortages and health inequities in various areas. This comprehensive review meticulously analyzes the literature and clinical trials of recent years, highlighting key AI models for DR diagnosis and treatment, including their theoretical bases, features, applicability, and addressing current challenges like bias, transparency, and ethics. It also presents a prospective outlook on the future development in this domain.

## 1 Introduction

In the prelude to our discourse, it is foretold that by the year 2045, the count of adults aged 20 to 79 afflicted with diabetes will soar to no less than 235 million souls, with the regions of greater structural poverty bearing the heaviest burden of both diagnosed and undetected cases (Joanne et al., 2012; Lm et al., 2013; Tan and Wong, 2022; Harreiter and Roden, 2023; Home, Resources, diabetes, L. with, Acknowledgement, FAQs, Contact, 2024). Diabetic retinopathy (DR), as the most common and fundamental ocular complication of diabetes, is projected to increase to a global burden of 160.5 million cases by 2,045 (Teo et al., 2021; Yao et al., 2024). It is a chronic eye disease influenced by a multitude of factors, often lurking in the shadows of the early non-proliferative stage without overt clinical symptoms, yet detectable through a series of examinations (Tien et al., 2009). As DR progresses, with the formation of neovascularization and other developments, it can lead to a cascade of complications, such as diabetic macular edema (DME) and other macular diseases, which are among the most severe vision-threatening conditions but not easily detected by machines in their early stages (Sundaram et al., 2023; Wu et al., 2024). To prevent irreversible vision loss, early detection and ongoing monitoring of diabetic retinopathy are extremely important (Sussman et al., 1982; Pedro et al., 2016; Wei and Acy, 2018; Resnikoff et al., 2020).

AI stands as a beacon of hope, adept at mitigating the challenges of substandard healthcare and scarce resources in regions where the incidence of DR runs rampant (Varadarajan et al., 2020; Dong et al., 2022). So far, AI models powered by machine learning and algorithms have been used to help diagnose and treat diabetic retinopathy. The models, combined with various diagnostics, offer increased sensitivity and specificity, greatly assisting doctors in DR detection and management. Yet, the journey is not without its trials; biases, cost-effectiveness, and transparency are but a few of the many hurdles that lay before us (Dsw et al., 2017; Andrzej et al., 2020; Anand et al., 2023).

**FIGURE 1:**
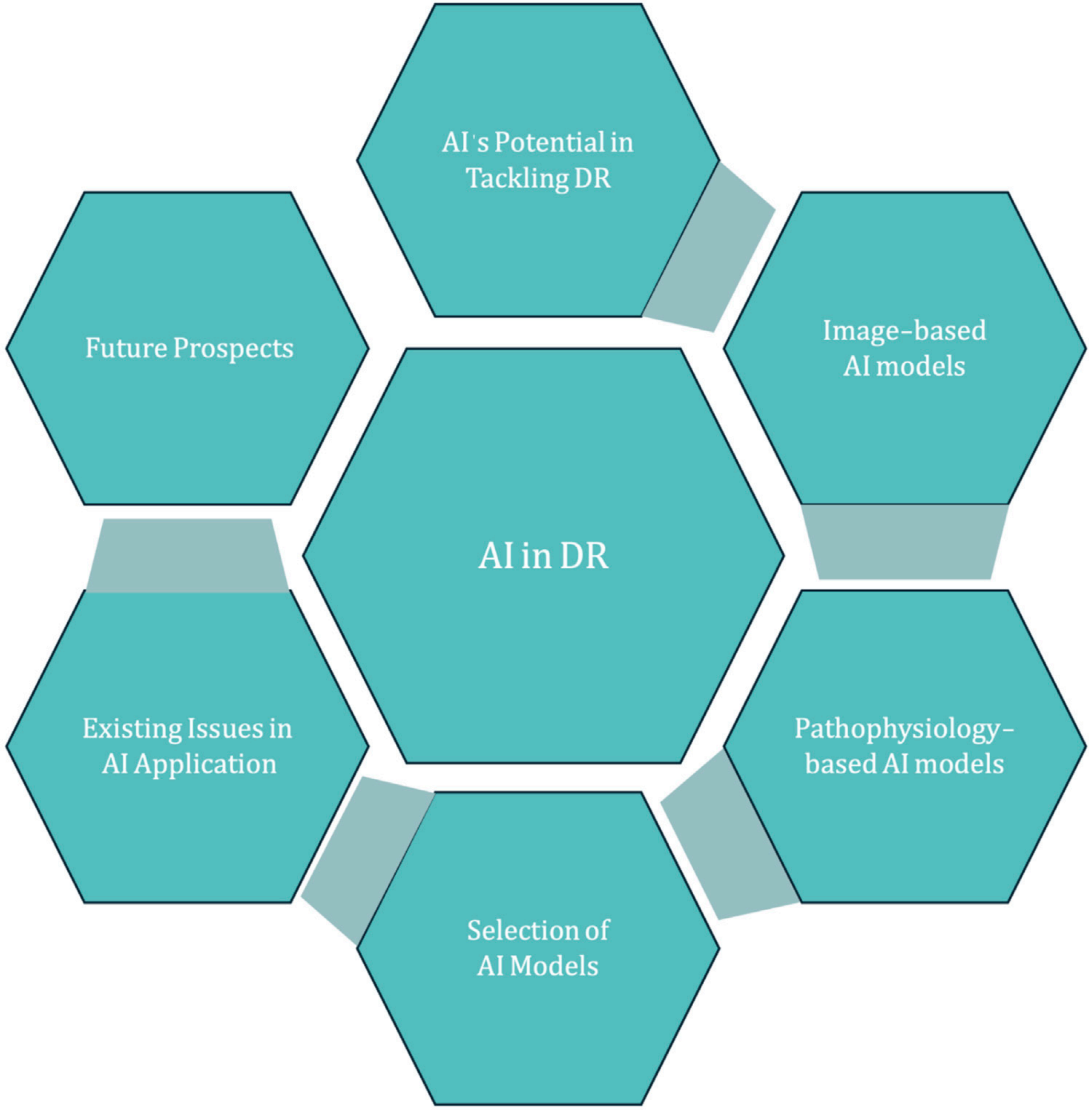
Introduction for AI in DR.

In this comprehensive treatise, we delve into the current state of AI in diabetic retinopathy diagnosis, treatment, and surveillance, with the goal of exposing existing shortcomings and mapping out a strategy for enhancement and creative advancement (Figure 1).

## 2 Applications of artificial intelligence in diabetic retinopathy

### 2.1 AI models based on imagistic output approaches

Diabetic retinopathy is characterized by a relatively clear chronic ocular fundus disease course (Table1, 2). Common methods for examining diabetic retinopathy include fundus photography, fluorescein angiography (FFA), optical coherence tomography (OCT), and even retinal smartphone photography is gradually being applied in the examination of diabetic retinopathy. AI models primarily diagnose the severity of diabetic retinopathy by analyzing the results of these examination methods, thereby suggesting corresponding treatment plans. AI assistance in diabetic retinopathy now also focuses on evaluating treatment, forecasting disease outcomes, and predicting the course of retinal lesions. Table 3 provides a detailed display of the advantages and disadvantages, applicability, and corresponding AI models used for these diagnostic methods.

**TABLE 1:** International clinical diabetic retinopathy severity grading criteria (Antonetti et al., 2012; Stitt et al., 2016).

Disease severity	Seen on examination of mydriatic eye disease
No significant DR	No abnormalities
Mild NPDR	Microaneurysms only
Moderate NPDR	Between mild NPDR and severe NPDR
Severe NPDR	Presence of any of the following changes but no signs of PDR: (1) more than 20 intraretinal hemorrhages in any of the four quadrants, (2) venous beading changes in 2 or more quadrants, (3) significant intraretinal microvascular abnormalities in at least one quadrant, and no changes in PDR
PDR	One or more of the following changes: (1) neovascularization (2) vitreous hemorrhage or preretinal hemorrhage

DR, diabetic retinopathy; NPDR, non-proliferative diabetic retinopathy; PDR, proliferative diabetic retinopathy.

**TABLE 2:** Stages of diabetic retinopathy in China (Xiaoxin, 2023).

Type	Stage	Seen on an eye examination
Simplicity	I	Have a micro-hemangioma or have a small hemorrhage
	Ⅱ	There is a yellow “hard oozing” or bleeding
	Ⅲ	There is a white “soft oozing” or bleeding
Proliferative	IV	There is neovascularization in the fundus or vitreous hemorrhage
	V	There is neovascularization and fibrous hyperplasia in the fundus
	VI	There is neovascularization and fibrous hyperplasia in the fundus, complicated by retinal detachment

**TABLE 3:** Characteristics and applications of imaging examination (Gulshan et al., 2016).

Imaging tests	Peculiarity	Suitable situations	Corresponding to AI models
Fundus photography	Non-invasive, most widely used and less costly; However, the field of view is limited, making it difficult to detect minor lesions	Initial screening, grading of diagnosis	IDx-DR, EyeArt, Retmarker, Google, SELENA, Bosch DR algorithm (Pritam et al., 2017), EasyDL, DeepDR (Ling et al., 2021), DeepDR Plus (Ling et al., 2024), Medios AI, PhelcomNet (Paisan et al., 2016; Hasan and Siddiqui, 2023)
FFA	Invasive, highly sensitive, easy to observe changes in microa and microvasculature, showing leakage; However, it is time-consuming, and the dye may cause adverse reactions	Screening for DR in tolerable populations, particularly for early lesions and observation of macular edema and leakage	ResNet18 models (Gao et al., 2023), etc., as well as unnamed models
Ultrasound	It is not commonly used and it is difficult to directly observe fundus lesions	Concurrent refractive interstitial opacity assists in the diagnosis of retinal detachment or hyperplastic traction	Not yet
OCT	It can quantitatively measure retinal thickness, check macular edema and epiretinal membrane, and cannot directly show vascular lesions, but can be stratified with AI	There are no significant PDR changes but significant visual acuity loss, mainly for DR macular degeneration	VGG16 CNN models (Ibrahim et al., 2020), CAD systems, etc
OCTA	Vascular details are visualized and quantified, but projection artifacts are formed	When retinal vascular lesions need to be analyzed in specific studies	VGG19 architecture (Le et al., 2020), etc.,
Ultra-wide-angle retinal imaging	High resolution, wide field of view; But the perimeter is distorted	Fundus examination	RETFound model, DeepUWF-Plus system (Liu et al., 2023), etc.,

FFA, fluorescein fundus angiography; OCT, optical coherence tomography; OCTA, optical coherence tomography angiography.

**FIGURE 2:**
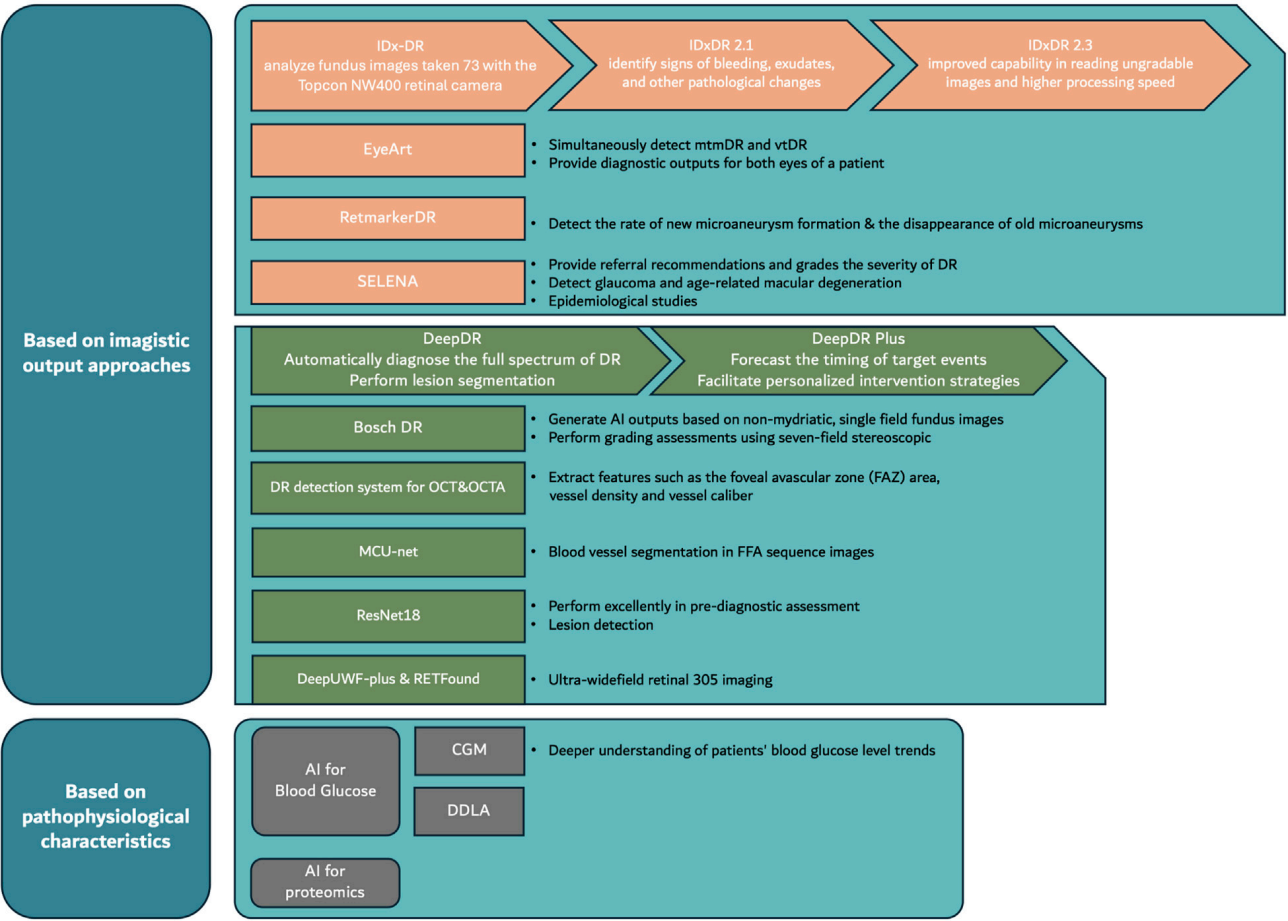
Main AI options in DR.

The main models and their characteristics are as follows (Figure 2).

#### 2.1.1 Fundus photography

Analyzing fundus photographs is one of the most advanced directions in DR AI models, with many approved or in-use models relying on this approach for diagnosis or staging. Here are the translations of some prominent models.

**FIGURE 3:**
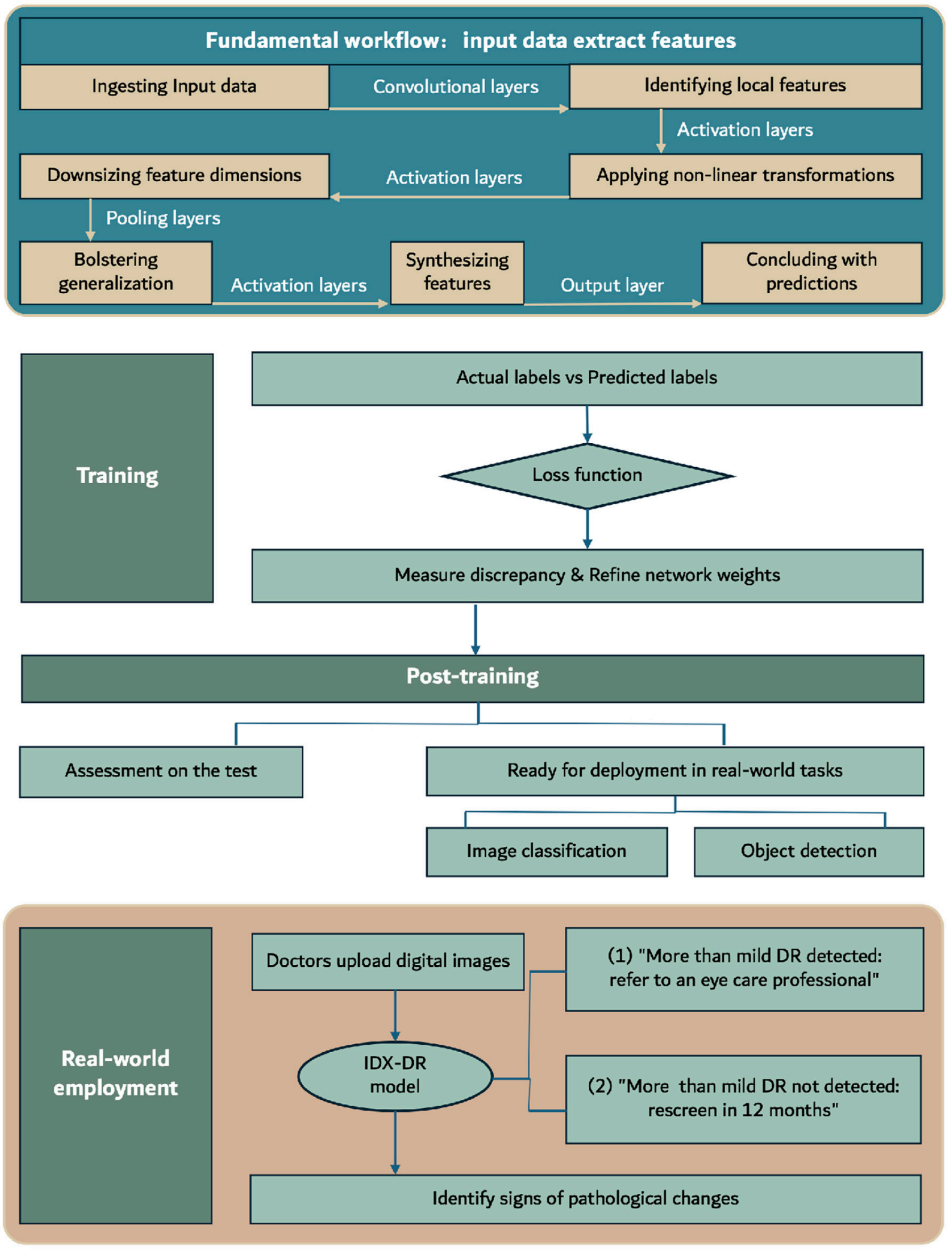
Steps for diagnosing DR with CNN.

IDx-DR, approved by the FDA in 2018, is the first medical device to use artificial intelligence to detect more than mild DR in adults with diabetes (Rajesh et al., 2023). This software utilizes deep learning algorithms, particularly convolutional neural networks, to analyze fundus images taken with the Topcon NW400 retinal camera (Andrzej et al., 2020; Commissioner, 2020). Convolutional Neural Networks (CNNs), a form of deep learning architecture, are extensively applied in areas like image and video recognition, as well as natural language processing. They adeptly extract features from input data through a structured sequence of layers. The fundamental workflow, as shown in Figure 3, encompasses: ingesting input data, identifying local features via convolutional layers, applying non-linear transformations with activation layers, downsizing feature dimensions through pooling layers, possibly bolstering generalization with normalization layers, synthesizing features in fully connected layers, and concluding with predictions made by the output layer. Throughout training, CNNs use loss functions to assess the difference between predicted and actual labels, adjusting weights through backpropagation. Optimization techniques such as gradient descent are employed to fine-tune parameters, ensuring consistent validation performance. Post-training, the model undergoes assessment on the test set and is subsequently ready for deployment in real-world visual tasks like image classification and object detection. The algorithm, trained on a large dataset of annotated images, can identify and classify retinal lesions such as microaneurysms, hemorrhages, and neovascularization. Doctors upload digital images of the patient’s retina to a cloud server running the IDx-DR software (Goldstein et al., 2023). If the image quality meets the standards, the software provides one of two results: 1) “More than mild DR detected: refer to an eye care professional” or 2) “More than mild DR not detected; rescreen in 12 months” (Commissioner, 2020). Compared to stringent human grading standards, the algorithm has a sensitivity of 87.2% and a specificity of 90.7%. Currently, the IDx-DR algorithm has been rebranded as LumineticsCore (Rajesh et al., 2023). In contrast to the IDx-DR 2.0, the IDx-DR X2.1 represents an advanced hybrid system that employs several Convolutional Neural Networks (CNNs). These networks are trained to identify signs of bleeding, exudates, and other pathological changes, in addition to the regular anatomy of the retina. They are seamlessly incorporated into a traditional framework akin to other prototype IDPs. The analytical software categorizes results into four distinct outputs: 1) Negative: suggesting an absence or only a mild degree of diabetic retinopathy (DR), 2) rDR: indicating the presence of referable diabetic retinopathy, 3) vtDR: indicating vision-threatening diabetic retinopathy, 4) Low image quality: signifying issues with the examination protocol or substandard image quality. This apparatus applies a suite of CNN-based detectors to each image captured during the examination. These detectors are meticulously trained and calibrated to discern both the normal anatomy, such as the optic disc and fovea, and the telltale signs of DR, including hemorrhages, exudates, and neovascularization (Michael et al., 2016). The latest FDA-approved version, IDx-DR 2.3, shows improved capability in reading ungradable images and higher processing speed compared to version 2.0, although the DR classification algorithm remains unchanged (Libres, 2024). However, its performance varies with different detection standards, primarily influenced by factors such as image quality, ethnicity, and the graders (Hansen et al., 2015; Andrzej et al., 2020).

Besides IDx-DR, EyeArt, as another extensively tested model approved by the FDA in 2020, is a deep learning-based classification tool developed by Eyenuk (Yingfeng et al., 2012). It is primarily designed for healthcare providers to automatically detect more than mild diabetic retinopathy (mtmDR) and vision-threatening diabetic retinopathy (vtDR) in adults who have not been previously diagnosed with these conditions (Businesswire, 2024). This detection is performed using fundus images taken with Canon CR-2 AF and Canon CR-2 Plus AF cameras. EyeArt is the first FDA-approved autonomous AI technology capable of simultaneously detecting mtmDR and vtDR in both primary care and eye care settings through a single test. Additionally, EyeArt is the first FDA-approved autonomous AI technology that can provide diagnostic outputs for both eyes of a patient. The accuracy and sensitivity of EyeArt have been subjects of debate. According to clinical trials conducted by the developers, EyeArt demonstrated a sensitivity of 96% and specificity of 88% for detecting mtmDR, and a sensitivity of 92% and specificity of 94% for detecting vtDR. All eyes with an ETDRS score of 43 or higher were accurately identified as mtmDR positive. In a clinical study conducted in the UK, EyeArt showed a sensitivity of 95.7% and a specificity of 54.0% (Heydon et al., 2021). In a prospective study in the US, the sensitivity for mtmDR was 95.5% and the specificity was 85.0% (Eli et al., 2021). In a recent community screening, the diagnostic accuracy was 81% (101/124 eyes), and the referral accuracy was 83% (103/124 eyes) (Vought et al., 2023). These results indicate that EyeArt’s performance can vary significantly depending on the tested population and reference standards.

In addition to EyeArt and IDx-DR, several other AI models have received Class IIa certification (CE Mark) in the European Union (EU), including Retmarker, Google, and the Singapore Eye Lesion Analyzer (SELENA) (Anand et al., 2023). Here are some brief introductions.

RetmarkerDR uses a feature-based machine learning model to screen for DR and detect biomarkers such as microaneurysms (MAs) (Retmarker, 2024b). It primarily relies on fundus images for diagnosis and is distinguished by its ability to compare current images with previous ones to assess disease progression (Andrzej et al., 2020). Although angiography is the gold standard for detecting microaneurysms, it is an invasive procedure that not all patients can tolerate. There is a significant correlation between MA turnover rates based on angiography and those based on retinal imaging (Rajesh et al., 2023). RetmarkerDR employs an advanced proprietary co-registration algorithm that automatically overlays retinal images and complements state-of-the-art MA detectors to calculate important rates related to MA turnover (formation and disappearance rates) (Haritoglou et al., 2014; Pappuru et al., 2019). This software can detect the rate of new microaneurysm formation and the disappearance of old microaneurysms, known as “microaneurysm turnover” (Pappuru et al., 2019; Li et al., 2019). This capability not only helps in detecting DR progression but also in evaluating the efficacy of treatments such as dexamethasone (Takamura et al., 2023). The specificity and sensitivity of RetmarkerDR’s early DR screening function have varied across different trials, but generally, both specificity and sensitivity have exceeded 90% (Sa, 2024a; Sa, 2024b; Retmarker, 2024a), although further research on its biomarker detection capabilities is needed.

Google developed a convolutional neural network-based algorithm for detecting DR in 2016, similar to IDX-DR (Gulshan et al., 2016). The algorithm was validated on multiple datasets, including EyePACS, Messidor-2, and a nationwide DR screening program in Thailand (Gulshan et al., 2019). On the Messidor-2 dataset, the algorithm achieved a sensitivity of 96.1% and a specificity of 93.9% in high-sensitivity settings, and a sensitivity of 87.0% and a specificity of 98.5% in high-specificity settings (Andrzej et al., 2020). In Thai primary care centers, the algorithm showed 91.4% sensitivity and 95.4% specificity in detecting severe non-proliferative DR, proliferative DR, or referable DME, surpassing a U.S. retinal specialist panel’s consensus (Paisan et al., 2022). User feedback suggests the AI system’s prompt results are helpful for referrals, yet uploading images can be difficult. Additionally, the algorithm occasionally deems images ungradable, requiring re-imaging or manual review (Ian Gerard et al., 2024).

SELENA, developed by the Singapore Eye Research Institute (SERI), is a deep learning-based system designed to enhance the efficiency of DR screening by analyzing fundus photographs (Gulshan et al., 2016). It also provides referral recommendations and grades the severity of DR. It was based on VGGNet, which is also a type of Convolutional Neural Network (CNN) like that of IDx-DR. The development of SELENA utilized multi-ethnic datasets to ensure its applicability across different ethnic groups (Wong et al., 2021). From (Wong et al., 2021), in primary validation datasets, SELENA demonstrated outstanding performance in detecting referable DR, with an AUC of 0.936, sensitivity of 90.5%, and specificity of 91.6%. For vision-threatening DR, SELENA achieved an AUC of 0.958, sensitivity of 100%, and specificity of 91.1%. Additionally, SELENA can detect glaucoma and age-related macular degeneration (AMD), showing high efficiency in these areas as well (Andrzej et al., 2020). To assess real-world viability, SELENA was evaluated in a mobile unit in Zambia, achieving an AUC of 0.973, 92.25% sensitivity, and 89.04% specificity for detecting referable DR, proving its effectiveness across different populations and limited-resource environments (Valentina et al., 2019). Furthermore, SELENA has shown excellent performance in epidemiological studies, significantly reducing the time and cost required for manual assessments. In one trial, SELENA was 360 times faster than manual evaluation, and the risk factors identified by SELENA, including diabetes duration, HbA1c levels, and systolic blood pressure, were consistent with those identified manually (Rajesh et al., 2023). The outcomes were comparable, underscoring SELENA’s strength in handling large datasets. The Singapore team intends to broaden SELENA’s use, integrating OCT for enhanced glaucoma detection and forecasting myopia, aiming for personalized healthcare (Wong et al., 2021).

Beyond the FDA and EU-approved models above, other promising models are yet to receive market release approval.

For instance, the Bosch DR algorithm can generate AI outputs based on non-mydriatic, single-field fundus images, as well as perform grading assessments using seven-field stereoscopic, mydriatic ETDRS imaging on the same eye (Pritam et al., 2017). EasyDL, developed by Baidu, utilizes the publicly available Kaggle diabetic retinopathy dataset and employs transfer learning techniques to establish an AI diagnostic model for fundus image analysis. It has demonstrated good performance in both training and testing sets, achieving an accuracy rate of over 91% and an AUC of 0.935 (Cao et al., 2022).

It is noteworthy that the model DeepDR along with its evolved version DeepDR Plus, can be considered a significant breakthrough in AI research for diabetic retinopathy (DR) lesions.

DeepDR, developed by Professor Jia Weiping’s team from the Department of Endocrinology and Metabolism at the Sixth People’s Hospital Affiliated to Shanghai Jiao Tong University, the Shanghai Diabetes Institute, and the Shanghai Diabetes Clinical Medical Center, is based on the world’s largest fundus image database. The system comprises three subsidiary networks: an image quality assessment network, a lesion detection network, and a diabetic retinopathy (DR) grading network. It employs a pre-trained model known as ResNet for feature extraction and leverages Mask-RCNN for task-specific architecture design, catering to image classification or segmentation tasks. By applying transfer learning techniques, DeepDR is fine-tuned on a labeled DR grading dataset, thereby enhancing its capabilities in lesion detection and segmentation performance. It employs a multi-task learning framework with enhanced transfer learning to automatically diagnose the full spectrum of DR, from mild to proliferative stages. Additionally, it provides real-time feedback on the quality of fundus images and performs lesion segmentation (Ling et al., 2021).

DeepDR Plus, a recent advancement in AI for DR diagnostics by Shanghai Jiao Tong University and Tsinghua University, has created an early warning system for DR complications. This system uses deep learning to analyze retinal image sequences and accurately predict DR progression, potentially informing new global screening and prevention strategies. From their new article (Ling et al., 2024), DeepDR Plus evolved from its predecessor, DeepDR, and has been developed, validated, and externally tested. The system encompasses a metadata model, a fundus image model, and a combined model. The fundus image model utilizes the ResNet-50 architecture as a feature extractor and enhances significant parts of the feature map through a self-attention mechanism. Initially, the system was pre-trained using 717,308 retinal images from 179,327 diabetic patients. Following this, a multiethnic dataset comprising 118,868 images of 29,868 diabetic patients was employed to train and validate the DeepDR Plus system. The system predicts event timings by estimating survival functions, using a fixed-size Weibull mixture model to simulate individual survival distributions. The deep learning network’s parameters are optimized by maximizing the likelihood function through self-supervised pre-training with MoCo v2, improving feature extraction. In predicting DR progression, the system achieved concordance indexes ranging from 0.754 to 0.846 and integrated Brier scores between 0.153 and 0.241 for all time points up to 5 years. The system’s validation in real-world studies showed that DeepDR Plus’s personalized screening intervals could extend the average interval to 31.97 months, from 12, and reduce the delayed detection rate of DR progression to 0.18%. This capability allows DeepDR Plus to support individualized risk assessment and management of DR by predicting the risk of DR progression within the next 5 years from a single retinal examination. The system can reduce unnecessary screenings, increase screening efficiency, potentially extend screening intervals, and minimize delayed detection of severe DR progression. Furthermore, its integration into clinical and digital workflows can facilitate personalized intervention strategies for the management of diabetic retinopathy.

Moreover, the process of uploading and processing images has become increasingly convenient and affordable. Systems such as Medios AI, EyeArt, and PhelcomNet have achieved high accuracy in diagnosing fundus images captured by smartphones (Pedro et al., 2016; Hasan and Siddiqui, 2023).

In summary, as the most mainstream and cost-effective method in current research, AI based on fundus photography has achieved many results in the early screening, prognosis, and disease progression monitoring of DR, but still needs to further improve its sensitivity and specificity.

#### 2.1.2 OCT and OCTA

Optical Coherence Tomography (OCT) serves as a primary diagnostic tool with high-quality equipment that makes it easy to detect any harmful changes. OCT is particularly effective for visualizing various retinal layers and has been extensively researched for diagnosing retinal diseases. For instance, by integrating features manually extracted from Regions of Interest (ROI) with the VGG16 Convolutional Neural Network (CNN) model, OCT imaging has been used to diagnose conditions such as macular edema, macular hole, central serous retinopathy, choroidal neovascularization and pigment epithelium detachment (Ibrahim et al., 2020). Many AI models based on OCT are primarily used for monitoring conditions such as edema and degeneration in the macula (Gulshan et al., 2019; Sheng et al., 2022; Lam et al., 2024). With the introduction of network for automatic quantification of macular edema (Tsuboi et al., 2023) and integration networks that segment multiple layers of the retina, the detection and grading of DR using OCT scans have achieved high accuracy and specificity (ElTanboly et al., 2017; Sandhu et al., 2018; Li et al., 2019; Ghazal et al., 2020).

A recent study (Elgafi et al., 2022) segmented retinal layers, extracted 3D features (including first-order reflectivity and the 3D thickness of each OCT layer), and employed a backpropagation neural network for classification. The results showed that the system achieved an accuracy of 96.81% using Leave-One-Subject-Out (LOSO) cross-validation. Integrating multi-layer features enhances DR detection accuracy. 3D features provide better lesion detail capture than 2D, surpassing single-feature and other ML methods.

Additionally, Optical Coherence Tomography Angiography (OCTA) can be used to identify retinal diseases. OCTA provides detailed views of the eye’s vasculature and retina. Research (Eladawi et al., 2018) has created a DR detection system utilizing OCTA images, extracting features like FAZ area, vessel density, and caliber through segmentation. These features were analyzed using a Support Vector Machine (SVM) classifier, achieving an accuracy of 94.3%, sensitivity of 97.9%, and specificity of 87%. Another study (Heisler et al., 2020) using integrated deep learning techniques trained a network on the VGG19 architecture, achieving a best accuracy of 92%, outperforming other architectures. Research (Le et al., 2020) using the VGG16 CNN architecture to diagnose DR with OCTA reported an accuracy, specificity, and sensitivity of 87.27%, 90.82%, and 83.76%, respectively.

Further studies have combined OCT and OCTA modalities to identify different levels of DR. For example, one study (Sandhu et al., 2020) used both OCTA and OCT to grade Non-Proliferative Diabetic Retinopathy (NPDR) in 111 patients. For OCTA, they extracted features such as vessel density, FAZ size, vessel caliber, and the number of intersections and bifurcations. For OCT, they performed retinal layer segmentation and extracted features from each layer, represented by thickness, reflectivity, and curvature. Finally, a Random Forest classifier was used for classification, achieving an accuracy of 96%, sensitivity of 100%, and specificity of 94%.

In summary, OCT offers high accuracy, particularly in cases where there is suspicion of a series of macular complications caused by vascular changes. However, due to its relatively higher cost and the already high accuracy of fundus photography, the widespread adoption of OCT models is comparatively limited.

#### 2.1.3 FFA

Fluorescein Fundus Angiography (FFA) is also a common method for fundus examination in detecting DR. Under an ophthalmoscope, what is deemed a “normal” fundus may reveal microaneurysms and microvascular changes during angiography. Early neovascular buds and areas of non-perfusion are not easily detectable with an ophthalmoscope, but they can be clearly observed through FFA, which is currently still the gold standard for retinal vasculature (Yang et al., 2022). However, due to its invasive nature and complexity, AI models using FFA for early DR screening are less common.

The earliest researchers proposed an automatic method for detecting Neovascularization of the Posterior Pole (NPA) in FFA images of patients with diabetic macular edema, laying the foundation for intelligent guidance in laser applications (Jin et al., 2020). Additionally, there is also research on a Multi-Path Cascade U-net (MCU-net) architecture for blood vessel segmentation in FFA sequence images, indicating its potential application in the quantitative analysis of vascular morphology in FFA sequence images (Gang et al., 2021; Ghassemi et al., 2021; Gao et al., 2023). Deep learning algorithms have been used to detect leakage points in Central Serous Chorioretinopathy (CSC) from dynamic FFA images (Chen et al., 2021).

In the most recent study (Gao et al., 2023), a research team collected and annotated a large number of FFA images, training a CNN model to recognize image quality, localization, eye side, imaging phase, and various lesions associated with DR. The results showed that the ResNet18 model performed excellently in pre-diagnostic assessment and lesion detection, with accuracy comparable to junior ophthalmologists. This study not only underscores AI’s potential to improve ophthalmic diagnostics but also suggests the model could enhance medical services, especially in resource-limited areas.

Despite some limitations, such as the singularity of the dataset source and the restriction of lesion types, the study lays the groundwork for future multi-center, multi-disease type FFA image analysis and automatic report generation.

In addition to AI models based on the aforementioned fundus photography, OCT, and FFA, alternative approaches include AI models for ultra-widefield retinal imaging, such as DeepUWF-plus and RETFound, which extend traditional fundus photography. However, these newer models lack extensive clinical trials. Moreover, there are hybrid approaches that combine one or two of the aforementioned techniques, like OCT and FFA. Due to limitations in length, these are not further discussed here.

### 2.2 AI model based on pathophysiological characteristics

In addition to diagnosing and treating DR through direct imaging of retinal lesions, AI can also be utilized based on other pathophysiological characteristics of DR. However, research in this area is relatively limited. These models support comprehensive and differential DR diagnosis from a mechanistic perspective. These models support comprehensive and differential DR diagnosis from a mechanistic perspective. This field warrants further research and investment.

#### 2.2.1 Blood glucose

Hyperglycemia, one of the primary causes of DR, is crucial for early screening and referral. Continuous glucose monitoring (CGM) systems, supported by portable devices, facilitate a deeper understanding of patients’ blood glucose level trends, including the amplitude, direction, timing, and frequency of glucose fluctuations (Jan et al., 2016). However, due to the multifactorial nature of DR, it is necessary to incorporate multiple metrics [such as Time in Range (TIR) and Mean Blood Glucose (MBG)] within CGM systems to accurately reflect the relationship between complications and CGM data (Irl, 2015; Yang et al., 2021).

Moreover, DR’s nonlinear nature challenges traditional risk models (Keith et al., 2014), a challenge that traditional risk prediction models cannot adequately address. To overcome the limitations of CGM data analysis in constructing risk forecasting models, researchers from the School of Information Science and Engineering at Northeastern University, in collaboration with the Sixth People’s Hospital of Shanghai Jiao Tong University and the Shanghai Diabetes Clinical Center, have proposed the consideration of deep learning techniques, which can effectively uncover nonlinear relationships (Tao et al., 2023).

In July 2023, they introduced a novel model known as the Double Deep Latent Autoencoder (DDLA) (Tao et al., 2024). From (Tao et al., 2024), this model uses CNN and Long Short-Term Memory (LSTM) networks to analyze time-series data, improving feature extraction and classification. The process involves feature extraction, handling missing data, feature fusion, and diagnosis through a fully connected network, culminating in the use of a softmax layer to output the most likely type of the current sample (either DR or non-DR in T2D patients). Preliminary results show an accuracy of 0.89 and a specificity of 0.97 for T2D patients. Further clinical trials are needed to validate its effectiveness in managing DR.

#### 2.2.2 Proteomics

DR is driven by multiple factors, including hyperglycemia, inflammation, and vascular dysfunction. Proteomic analysis can identify various cell-specific protein markers involved in these processes, providing auxiliary insights into the cellular drivers of DR and their roles at different disease stages (Wolf et al., 2023). Initially, aqueous humor (AH) and vitreous fluid samples are collected from patients for liquid biopsy (Gold et al., 2010). High-resolution proteomics via aptamer assays identifies numerous proteins, which are then integrated with scRNA-seq data from diverse ocular and extraocular cells (Tavé van et al., 2020; Joseph et al., 2021; Pradeep et al., 2021; Wolf et al., 2022; Wolf et al., 2023). This integration allows for the identification of the detected proteins’ cellular origins, thereby pinpointing markers unique to specific cells. AI models can extract local spatial features and global temporal dependencies, enabling the prediction of disease progression.

From Wolf et al. (2023) we can see that in the aqueous fluid of individuals with DR, changes in the expression of marker proteins from immune cells (B cells, macrophages, neutrophils, T cells, and mast cells), vascular endothelial cells, pericytes, and retinal glial cells, as well as liver proteins, have been observed. Studies have identified liver-derived proteins in DR as being associated with the acute phase response, acute inflammatory response, coagulation, and wound healing (Wolf et al., 2023). Immune cells, particularly macrophages, play a key role in the progression of late-stage DR in humans (Lee et al., 2015). Regarding the key factor of neovascularization used for staging DR, 58 angiogenic proteins showed different expression levels in DR. Twenty-nine of these proteins were elevated in both stages of the disease, whereas the remaining 29 were uniquely elevated in PDR and not in NPDR. Detecting these proteins through the model helps provide a comprehensive understanding of the current stage of DR. Additionally, therapeutic targets such as VEGFA for both PDR and NPDR can help guide more targeted treatment strategies.

In summary, this model provides a comprehensive risk assessment, distinguishing between NPDR and PDR based on the expression of angiogenic proteins and other markers. This approach provides a thorough molecular risk assessment for DR and aids in detecting other diseases for differential diagnosis and broad screening. Current research in this area is still relatively limited, warranting further attention and investment. In addition to these markers used in AI models, numerous studies are currently exploring similar markers. For instance, we can analyze angiogenic properties through core proteins and proteoglycans to predict the efficacy of anti-VEGF drugs (Low et al., 2021), all of which could be incorporated into future AI models for predicting and monitoring disease progression and treatment status.

The aforementioned models primarily focus on analyzing various retinal surface or cross-sectional images, which generally have inherent limitations. Firstly, building a large and diverse database is essential for the model’s broad training and applicability. Sample diversity, including ethnic variations, image clarity, and pupil dilation status, is critical for ensuring model accuracy and generalization to new images.

In practical applications, to enhance the model’s accuracy and reliability, choose AI models trained on similar databases or adjust input image resolution, capture conditions, and parameters to match the training data.

Furthermore, the model’s interpretability and robustness should be considered, ensuring that the model can still provide stable and accurate analytical results in the face of poor image quality or noise. This may necessitate further algorithm optimization and model tuning to enhance the model’s adaptability to various image variations.

## 3 Prospects and challenges of AI in the diagnosis of DR

### 3.1 AI technology issues and challenges

#### 3.1.1 Bias and prejudice issues

AI algorithms necessarily undergo pre-training and training through databases, which may involve statistical methods (Valentina et al., 2019). Since the essence of a database is a sample derived from the population, it can possess all kinds of biases that can occur in samples and statistical processes, such as selection bias and confounding bias, etc., (Sheng et al., 2022). To boost the algorithm’s accuracy, we need to expand the dataset, diversify sample types, and adjust the algorithm for optimal sample sizes. Also, integrating multi-parameter and regression analyses, as well as managing standardized variables and parameters, is crucial. Addressing algorithmic biases, such as those from dataset imbalances, requires more and varied samples (Popescu Patoni et al., 2023). Feature selection bias mainly occurs because some important clinical indicators are ignored in the feature extraction, which requires cooperation with clinical doctors to comprehensively enter or select the most symbolic reference indicators (Sheng et al., 2022). The algorithm excels on familiar datasets but struggles with new ones, highlighting the need for continuous training to improve its generalization (Poly et al., 2023). In the improvement of algorithms, the “black box” poses a substantial challenge. AI models, especially deep learning models, are usually inexplicable to the outside world, and their decision-making process lacks transparency (Dey et al., 2022). This implies that while these models can precisely detect image patterns and diagnose, their reasoning process remains unclear. This lack of transparency makes it hard for users, including doctors and patients, to grasp the model’s mechanisms, limiting deep understanding, utilization, and enhancement of the AI. It also complicates the oversight of the machine’s decision-making, affecting its accountability and verifiability (Ghassemi et al., 2021). To solve these problems, researchers and developers are exploring explainable AI (XAI), local interpretation, model simplification, etc., At the same time, the government and related departments also need to formulate relevant policies and regulations, requiring AI systems to provide a certain degree of transparency and interpretability (Muhammad and Bendechache, 2024). A range of strategies is required to establish a rigorous and orderly AI environment.

#### 3.1.2 More development directions of AI

Multimodal imaging, combining data from various imaging modalities, will be an important area of growth, providing richer data for AI analysis (Zhenwei et al., 2024). Integrating various examination results such as OCT, FFA, and fundus photography into comprehensive analysis will offer a more detailed perspective on retinal conditions and improve the sensitivity and specificity of diagnosis (Xinyu et al., 2023). AI can analyze big data by integrating various factors like imaging with blood sugar levels and risk factors, using deep learning to identify image features such as vascular abnormalities, exudation, retinal edema, etc., to jointly calculate the DR risk probability. AI algorithms enhance analysis accuracy by preprocessing images through noise reduction, contrast adjustment, and normalization (Moor et al., 2023). Additionally, they utilize multimodal data to predict the progression of DR, patient responses to treatment, and assess the risk of other difficult-to-detect diabetes-related complications, such as diabetic nephropathy, which are based on microvascular lesions like DR (Lakshminarayanan et al., 2021). We can develop AI models capable of identify patterns of comorbidities such as diabetic nephropathy and cardiovascular diseases. In summary, there are too many areas worth improving and innovating.

### 3.2 Clinical improvement

In advancing AI technology, the collaboration between computer professionals and clinical doctors is crucial. Clinicians play a significant role, as many AI models are tested by comparing their performance against doctors’ diagnoses, using existing cases as training data (Nakayama et al., 2023). The accuracy of these models, including their specificity and sensitivity, is often evaluated based on clinician outcomes, which directly impacts test effectiveness. While the primary DR grading system follows international standards, if these do not support effective AI training, alternative grading methods can be considered (Gulshan et al., 2016). Moreover, AI should enhance DR diagnosis and monitoring, particularly by refining the referral process upon detecting suspicious lesions, to leverage AI’s potential for efficient screening. Additionally, Though there is no definitive evidence that different diabetes types cause unique retinopathy symptoms, some studies have shown that they may have different incidence rates, disease courses, pathogenic mechanisms, metabolomic characteristics, variable response to treatments (Romero-Aroca et al., 2017; Eid et al., 2019; Matuszewski et al., 2020; Nakayama et al., 2021). The need for AI model design to distinguish between types like Type 1 and Type 2 diabetes for detailed analysis is an important area for healthcare professionals to advance AI in clinical settings. Continuously refining DR diagnostic methods is essential for enhancing its detection rate. Additionally, we need to focus more on pathophysiological and biochemical mechanisms at the molecular level to discover more biomarkers for diagnosis and prediction. All of these require ophthalmologists, endocrinologists, and radiologists to work together to find appropriate solutions.

### 3.3 Selection of AI model

Once an AI model is created, we must not only know how to improve it but also how to utilize it effectively. When selecting a model, fully consider the actual situation. For example, in the early stages of diabetes, if resources and patient health allow, an AI model based on FFA (Joseph et al., 2021) can be chosen for its accuracy. However, for convenience and non-invasive screening, an AI model based on fundus photography might be more appropriate (Lam et al., 2024). If you want to monitor the course of DR development, you can use the Singapore model SELENA (Wong et al., 2021) or the DeepDR Plus (Lam et al., 2024) from China. And if you want to make a differential diagnosis or evaluate the effectiveness of treatment targets in the case of multiple eye diseases, you can use molecular diagnostic models (Wolf et al., 2022). For long-term screening and management of early and mild cases of diabetes, you can first use a DR diagnostic model based on (Liu et al., 2023) for screening and then refer.

### 3.4 Cost-effectiveness solution of AI model research

AI in DR diagnosis streamlines preliminary screening for doctors, particularly useful for high-volume medical facilities (Chung et al., 2024). It automates and continuously performs preliminary diagnostic tasks, ensuring accuracy and sensitivity, reducing misdiagnosis and related medical costs (Chung et al., 2024). Additionally, AI enables telemedicine, benefiting remote patients by reducing travel expenses and time (Gunasekeran et al., 2020).

However, despite the long-term cost savings, the initial investment in AI systems can be substantial. This includes software licensing, hardware procurement (such as high-performance computing resources), and professional training costs (Nakayama et al., 2023). Besides this, AI models must regularly incorporate new medical findings and data, necessitating ongoing research and development, maintenance, and upgrades to maintain their precision and efficacy (Ruamviboonsuk et al., 2021).

Research suggests that in DR screening, the most cost-effective approach is a semi-automated system where AI performs an initial assessment, followed by human review of DR-positive cases. However, in low-income regions, where AI screening is most needed, the cost-effectiveness of AI may be diminished by technology investment challenges, hindering AI adoption (Cleland et al., 2023). Addressing the affordability of AI is a critical issue that must not be overlooked.

### 3.5 Ethical issues

Beyond technical and efficacy concerns, ethical issues also merit attention. This includes data privacy and security, the “black box” challenges of technical safety and responsibility, trust in AI by both medical professionals and patients, and algorithmic bias (Abdullah et al., 2021). These are all significant considerations in the AI realm.

## 4 Conclusion

After an integrated analysis of the current literature and clinical trials, we have reached the conclusion that AI for DR diagnostics is evolving positively, enhancing diagnostic efficiency and accuracy while easing the workload and facilitating remote healthcare. Yet, challenges and opportunities lie ahead. To propel the further development of AI in DR treatment, we must foster interdisciplinary cooperation to innovate and integrate AI into clinical practice, addressing ethical, legal, and trust issues to ensure AI genuinely aids medical professionals and serves the wellbeing of the populace.
